# Comparing Participant Experiences of at‐Home and Hospital‐Based Biological Sampling: Cross‐Sectional Insights From the SIREN Study

**DOI:** 10.1002/hsr2.71199

**Published:** 2025-09-29

**Authors:** Irina Lut, Sarah Foulkes, Amanda Henry, Sophie Russell, Nipunadi Hettiarachchi, Jasmin Islam, Ana Atti, Susan Hopkins, Victoria Hall

**Affiliations:** ^1^ UK Health Security Agency London UK

## Abstract

Engaging and retaining research participants in studies that require sampling (e.g., blood, sputum) can be challenging. Regularly contributing biological sampling can be demanding for healthcare workers (HCW) in particular. SIREN is a prospective cohort of HCW in the UK who have been carrying out COVID‐19 testing since 2020. We aimed to evaluate satisfaction with at‐home PCR and blood sampling by collecting SIREN participants' feedback regarding sampling processes for COVID‐19 testing. We explored the acceptability of at‐home (PCR swab and finger‐prick blood sampling) compared to at‐hospital (PCR swab and phlebotomy) sampling. Thematic analysis was used to code free‐text responses. Out of 2,816 respondents, 74% preferred PCR testing at home compared to on site. Half of 1,279 participants who returned blood samples using a postal kit preferred to complete serological sampling at home instead of in hospital (52%). One in five reported no preference. Participants valued the ease and convenience of home‐sampling and clear communication about instructions and test results. Some participants reported difficulties with blood collection or logistic issues related to kits, but this did not prevent them from returning samples nor deter them from undergoing sampling in future research. Home‐sampling for PCR and serological testing was acceptable and feasible in this HCW cohort. Self‐sampling can be a cost‐effective and efficient way of collecting participant data. Clear communications about instructions for sample collection and the purpose of capturing the sample, easy‐to‐use devices and ensuring participants feel valued are strong facilitators to high uptake, and on‐going study retention.

## 1 Introduction

There are known barriers to engaging and retaining research participants in studies that require sampling (e.g., blood, sputum), including the time commitment associated with the collection, complicated sample collection protocols, and concerns about confidentiality or how their data may be used [1, 2]. Healthcare workers (HCW) in particular experience a physically and mentally demanding workload paired with complex work schedules, which can affect their capacity to regularly contribute biological sampling at their place of work or alternative study site. Participant‐centred approaches like the use of home‐sampling and micro‐sampling devices have been shown to aid in overcoming some of these challenges for diagnostic testing, chronic care provision, and clinical trial research [3, 4, 5, 6].

SIREN is a prospective cohort of HCW in the UK who have been carrying out COVID‐19 testing since 2020. To maintain frequent and large‐scale testing while reducing study costs, SIREN transitioned away from exclusively hospital‐based sampling by introducing self‐sampling for a subset of participants. We aimed to evaluate satisfaction with at‐home PCR and blood sampling by collecting SIREN participants' feedback regarding sampling processes for COVID‐19 testing.

## 2 Methods

In April 2024, 5710 participants who remained enrolled in SIREN until 31st March were invited to complete a structured online feedback survey regarding their experiences of taking part in data collection from September 2023 to March 2024. During the study period, swabs for PCR testing were collected fortnightly and blood samples for serology testing were collected every 2 months [7]. Serological sampling took place through a site‐based pathway with a phlebotomist in hospital or a postal pathway with a home‐based finger‐prick kit [8]. For PCR testing, participants were asked to provide samples from both the nose and throat, simultaneously. Participants undergoing any type of self‐sampling were sent kits to their home along with pre‐printed return labels and an instruction sheet with information on how to collect the sample and repackage it for return.

Participants had previously consented into data collection within the SIREN Study, including any surveys. We collected insights on the acceptability of at‐home (PCR swab and finger‐prick blood sampling) compared to at‐hospital (PCR swab and phlebotomy) sampling, with both dichotomous and Likert scale response options. Participants could additionally provide free‐text information on their experiences of at‐home testing.

Proportions were calculated for each survey response. Thematic analysis was used to code free‐text responses into themes and sub‐themes, which were identified by one researcher (I.L.). Responses were coded into a maximum of two themes as appropriate. All coding was also checked by a second researcher (A.H.). Coding discrepancies were discussed until consensus was achieved, and themes were finalised with input from the rest of the study team.

This sub‐study was approved by the Berkshire Research Ethics Committee (IRAS ID 284460, REC Reference 20SC0230) on 14 November, 2022.

## 3 Results

The survey response rate was 49% (2816/5710). All respondents had experienced site‐based sampling within SIREN, and 45% (1279/2816) additionally used a capillary sampling method (finger‐prick) for blood sampling at home. Out of 2816 respondents, 74% of participants preferred PCR testing at home, compared to 6% who preferred on‐site and 20% who reported no preference.

**Figure 1:**
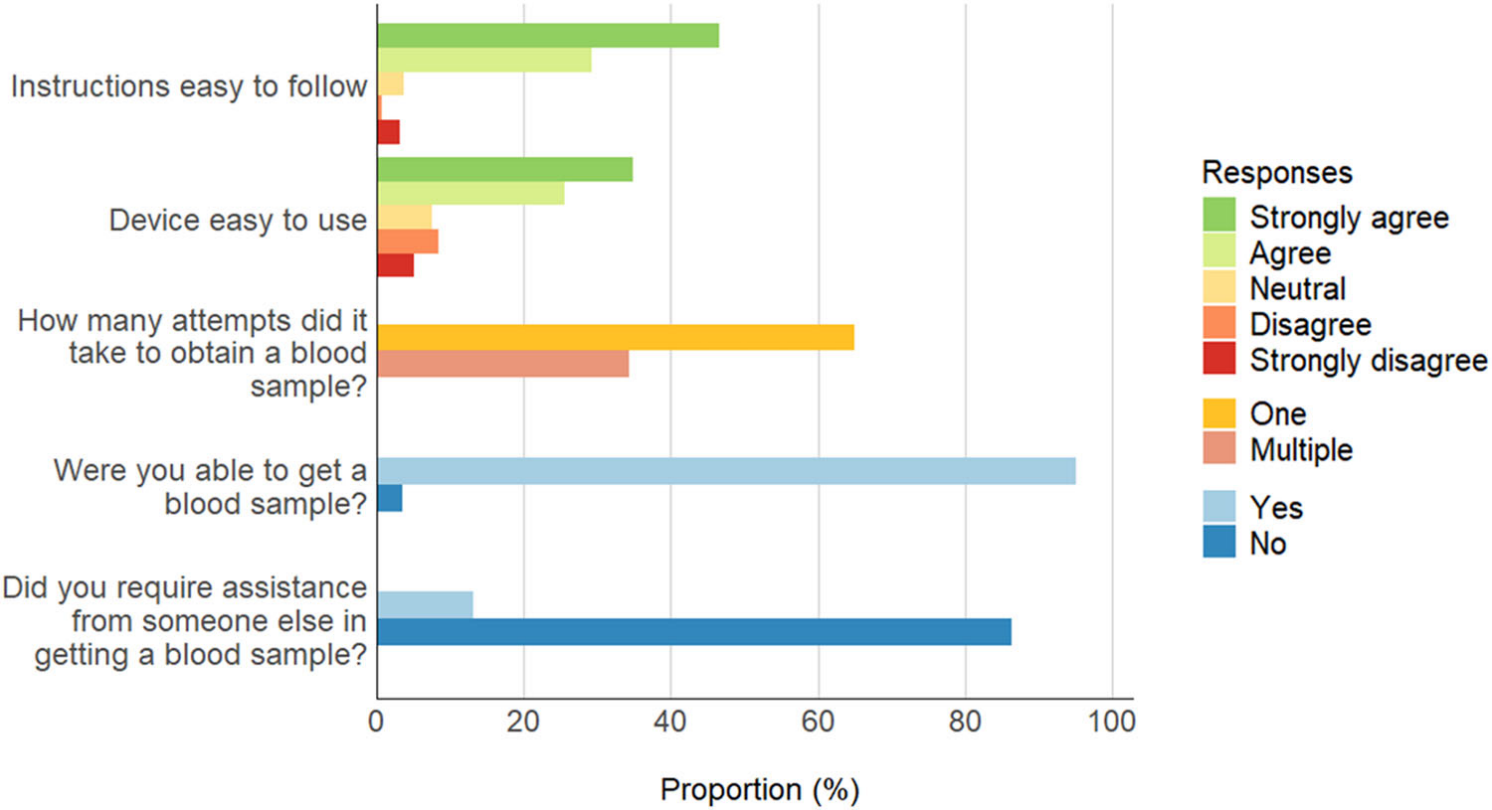
Feasibility of using postal serology kits for SIREN 2.0 testing.

Half of the 1279 participants who returned blood samples using a postal kit preferred to complete serological sampling at home instead of in hospital (52%), compared to 26% who preferred on‐site and 22% who reported no preference. The majority of participants who returned blood samples agreed or strongly agreed that the device was easy to use (61%). Almost all (95%) were able to obtain a blood sample, and the majority did so without requiring assistance (86%) (Figure 1).

In addition to quantitative responses, 611 (22%) participants provided additional information within a free‐text box. Five key themes demonstrate the positive and negative aspects of participants' home‐sampling experiences (Table 1). Positive themes highlight the ease and convenience of home‐sampling and clear communication about instructions and test results. Negative themes comprise issues with sample collection itself, logistics of receiving and returning kits, and suggestions on how communication could be further improved. Free‐text responses, for both PCR and blood sampling, indicated that participants felt the at‐home option was simple and more suited to their personal and professional schedules than going on site. Use of at‐home PCR swabs was well received with limited negative feedback.

**Table 1:** Themes and sub‐themes from free‐text survey responses about SIREN 2.0 home‐sampling.

PCR				
Themes		Sub‐themes	Participants (*n*)	Example quotes
Easy and convenient			351	*“A lot easier to receive at home and do in own time […]. Having PCR swabs arrive in post was a good aide memoir to complete!”*
Communications		Clear instructions and reminders	25	*“Instructions were clear and it was simple to do.”*
		Informative	57	*“PCR testing gives me peace of mind.”*
Sample collection issues		Throat swabs uncomfortable	9	*“I struggled to swab the back of the throat as I have a strong gag reflex”*
		Tubes/kit difficult to use	38	*“The tube tops were a bit difficult to close with swabs, but I managed.”*
Logistics		Questionnaire timing or comprehension	26	*“When sending the questionnaire please think about sending it in advance.”*
		Kits	33	*“On a few occasions, kits arrived home at the last minute.”*
		Prefer workplace	18	*“I never minded doing it at home, but I felt that I never completed it in a timely enough manner. I found it better having the tests done on site.”*
Communications			18	*“It is helpful to get text reminders and links and not just emails.”*

Serology				
No issues with sampling	Easy and convenient		99	*“Easy to use and having only to do a finger prick is much easier than having a blood test from your vein.”*
	Acceptable but learning curve		58	*“Blood collection was a bit challenging, but I always managed to get a sample.”*
Communications	Clear instructions and reminders		14	*“Instructions have always been clear […] and communication always open for questions.”*
	Informative		22	*“Good to know I still have antibodies […] regular testing […] has been good when visiting elderly patients and family.”*
Sample collection issues	Prefer on‐site		59	*“I prefer venous collection over finger/capillary collection if possible.”*
	Difficulty using kit components		206	*“Had to do this multiple times, leaving your fingers sore for a few days.”*
Logistics			14	*“I find that if I could bring the testing kits into work rather than going to a specialist postbox would make this process much easier.”*
Communications			23	*“I was not clear about when to take PCRs and blood samples as there was some delays between emails and parcels on the post.”*

One in six participants (17%) who used the at‐home kit for blood sampling reported issues, which included pain and sore fingers, difficulty obtaining sufficient blood, and problems with the vial being too narrow and difficult to manipulate. Importantly, reported difficulties with blood collection or logistic issues related to kits did not prevent participants from returning samples and would not deter them from undergoing sampling in future research. Most participants reported willingness to undertake at‐home PCR testing (96%) and at‐home blood sampling (82%) for future research.

## 4 Discussion

We found that home‐sampling for PCR and serological testing was acceptable and feasible in this HCW cohort. This is in line with other studies that showed that home‐based sampling of saliva and nasal fluid is acceptable for longitudinal microbial surveillance in both adults and children in the UK [9]. Our results further replicate findings from a study on at‐home blood self‐sampling for rheumatology patients, which found that facilitating increased independence for participants and using flexible methods which save time, improved monitoring compared to blood collection in a hospital [10]. A key strength of our study relative to other published work is the large sample size and inclusion of both PCR and blood samples.

Self‐sampling can be a cost‐effective and efficient way of collecting participant data. Our results indicate the implementation of self‐sampling at home is a successful way to continue ongoing collection of longitudinal samples. Furthermore, the SIREN experience indicates that participants are amenable to changes in sampling methods while the study is ongoing. This analysis is based on a large sample size and qualitative responses provide meaningful context and nuance to the proportions presented.

It should be noted that survey respondents compared their experiences of site‐based sampling which occurred from 2020 to 2022, with at‐home sampling which occurred in 2024. Therefore, respondents' views could be subject to recall bias. Furthermore, it is not clear if our findings represent the views of all clinical research study populations. This cohort of healthcare workers may have different perceptions and degrees of familiarity around blood sampling methods compared to other research participants.

While there are clear benefits to using self‐sampling methods for data collection, researchers should ensure that a switch in methodology from hospital‐based to at‐home sampling does not jeopardise the quality of samples collected and ensure that study objectives are still met. Providing opportunities for continual feedback from participants is important when introducing changes to sampling methods to identify factors that could negatively impact participant retention over time.

## 5 Conclusion

The experiences of SIREN participants who used both hospital‐based and at‐home sampling will inform future data collection and participant engagement to ensure a valuable and accessible involvement in research. Clear communications about instructions for sample collection and the purpose of capturing the sample, easy‐to‐use devices, and ensuring participants feel valued are strong facilitators to high uptake, and on‐going study retention.

## Author Contributions

**Irina Lut:** writing – original draft, writing – review and editing, formal analysis, methodology, validation, and data curation. **Sarah Foulkes:** writing – review and editing, supervision, formal analysis, methodology, conceptualization, and visualization. **Amanda Henry:** writing – review and editing, formal analysis, and validation. **Sophie Russell:** writing – review and editing, formal analysis, and project administration. **Nipunadi Hettiarachchi:** writing – review and editing, and writing – original draft. **Jasmin Islam:** writing – review and editing, and supervision. **Ana Atti:** writing – review and editing, supervision, methodology, conceptualization, and formal analysis. **Susan Hopkins:** writing – review and editing, supervision, and funding acquisition. **Victoria Hall:** writing – review and editing, supervision, conceptualization, and funding acquisition.

## Conflicts of Interest

The authors declare no conflicts of interest.

## Transparency Statement

The lead author, Irina Lut, affirms that this manuscript is an honest, accurate, and transparent account of the study being reported; that no important aspects of the study have been omitted; and that any discrepancies from the study as planned (and, if relevant, registered) have been explained.

## Data Availability

The data that support the findings of this study are available on request from the corresponding author. The data are not publicly available due to privacy or ethical restrictions.
